# Feline hippocampal and piriform lobe necrosis as a consequence of severe cluster seizures in two cats in Finland

**DOI:** 10.1186/s13028-015-0127-x

**Published:** 2015-07-28

**Authors:** Sara Fors, Sofie Van Meervenne, Janis Jeserevics, Mindaugas Rakauskas, Sigitas Cizinauskas

**Affiliations:** AniCura Regiondjursjukhuset Bagarmossen, Ljusnevägen 17, SE-128 48 Bagarmossen, Sweden; AniCura Läckeby Djursjukhus, Örntorp 201, SE-39598 Läckeby, Sweden; Referral Animal Neurology Hospital Aisti, Virtatie 9, 01600 Vantaa, Finland

**Keywords:** Epilepsy, Cat, Hippocampal necrosis, Seizure

## Abstract

Feline hippocampal and piriform lobe necrosis (FHN) has been reported from several countries worldwide and is considered an important aetiology for feline epileptic seizures. The aetiology of FHN remains unclear, however it is suspected that FHN might occur secondary to intense epileptic activity as described in humans and dogs although this has not yet been documented in cats. The purpose of our report is to describe the first cases of FHN in Finland diagnosed by magnetic resonance imaging (MRI) and histopathology. The two cases we describe had a well documented history of pre-existing seizures with normal brain MRI at the onset of cluster seizures but MRI done when the cats exhibited clinical deterioration secondary to severe seizure activity, revealed lesions in the hippocampus and piriform lobes typical of FHN. Our report confirms that feline hippocampus and piriform lobe necrosis does occur in the Finnish cat population and should therefore be considered as a differential diagnosis in cats with seizures. In addition, the presentation, clinical findings, results of MRI and/or histopathology shows that cats may develop FHN secondary to severe seizure activity.

## Background

Feline hippocampal necrosis (FHN), also known as necrosis of the hippocampus and piriform lobe, is recognized in several countries worldwide, with reports from Switzerland, Italy, Austria, United Kingdom, USA and Australia among others, and is considered an important aetiology for seizures [1–7], especially in feline complex partial seizures with orofacial automatism associated with hippocampal necrosis (FEPSO-HN) [8]. Interictal behavioural changes are regularly seen in addition to the seizures [1–4, 8]. A presumptive diagnosis of feline hippocampal necrosis can be made with magnetic resonance imaging (MRI) in vivo [3, 4, 8, 9] and is characterized by bilateral lesions in the hippocampus and occasionally in the piriform lobe, and the final diagnosis is confirmed by histopathology; with lesions consisting of acute neuronal degeneration and evolving to complete necrosis mainly in the pyramidal layer and occasionally the dentate gyrus of the hippocampus [1–3].

Feline hippocampal necrosis appears to have a diverse aetiology, with a presumable environmental or toxic aetiology suspected in early reports [1–3]. Later reports have described FHN as a consequence of auto-immune inflammation (limbic encephalitis) [9], together with neoplasia, a so called dual-pathology [10] and vascular aetiology has been suggested [11, 12]. Recently, FHN as a consequence of seizures has been hypothesized [5, 8].

The first purpose of this retrospective case study is to demonstrate that FHN occurs in Finland. The second purpose is to report that cats with hippocampal lesions typical for FHN at necropsy and/or MRI can have a longer history of seizures with normal MRI at the onset of cluster seizures, indicating that FHN may develop secondary to severe seizure activity in cats.

## Case presentations

The cases were collected retrospectively from medical records. Two male cats were examined at Referral Animal Neurology Hospital Aisti, Finland. Both cases were examined by a diplomate or a resident of the European College of Veterinary Neurology (ECVN). Both cats were native to Finland, had limited or no outdoor access and in one case other cats in the same household remained healthy throughout the observation period. Details regarding ancillary diagnostics, emergency treatment, at home treatment, anaesthesia, MRI equipment, positioning, sequences, imaging parameters and contrast media not presented in the text can be found in Tables 1 and 2.

**Table 1:** Summary of selected material, methods and results not mentioned in the text for two cats with FHN

	Case 1	Case 2
Ancillary diagnostics	Haematology and serum biochemistry profile normal, CSF normal	Haematology and serum biochemistry profile normal, EEG^d^-no epileptic activity
	EEG^d^ (2nd exam): duration 39 min 55 s; continuous short paroxysms and spikes at the C3 electrode	
Emergency treatment	2nd exam: medetomidineª 75 µg/kg	1st exam: phenobarbital^b^ 10 + 4 + 4 mg/kg IV, diazepam^c^ 0.5 mg/kg IV, medetomidine^a^ 40 µg/kg intramuscular
	IM, phenobarbital^b^ 10 mg/kg IV	2nd exam: medetomidine^a^ 30 µg/kg IM, diazepam^c^ 0.5 mg/kg IV and phenobarbital^b^ 6 mg/kg IV
At home treatment	Diazepam 0.6 mg/kg twice daily (BID) orally (PO) was started the day prior to referral	Phenobarbital^b^ 3 mg/kg BID PO prior to referral
	Phenobarbital^b^ 1.9 mg/kg BID PO, increased to 3.8 mg/kg BID after 2 months	

*EEG* electroencephalography, *IV* intravenously, *IM* intramuscularly, *PO* per os, *SID* once daily, *BID* twice daily.

^a^Domitor vet, Orion Pharma Animal Health, Finland.

^b^FenemalRecip, Recip, Sweden; Barbivet^®^, Vetcare oy, Finland.

^c^Stesolid^®^ Novum, Actavis, Iceland.

^d^EEG performed using portable EBNeuro EEG equipment (Galileo Be Light Peripheral Configuration, EBNeuro, Firenze, Italy) and a method of standardized placement of EEG electrodes resembling the 10–20 international system for humans. A 14-channel reference montage (F7, F3, F4, F8, T3, C3, Cz, C4, T4, P3, Pz, P4, O1, O2; sensitivity, 10 µV/mm; time constant, 0.3 s; Hf, 70 Hz; notch filter inserted; reference: between the eyes on the ridge of the nose; ground: caudally to the external occipital protuberance) was used to record the EEG. Sixteen EEG needles (EEG needles, 30 gauge 15 mm monopolar stainless steel needle electrodes, BIONEN S.a.s., Firenze, Italy) were inserted as subdermal active, reference, and ground electrodes. Impedances did not exceed 5 Ω.

**Table 2:** Summary of methods: anaesthesia, MRI sequences, imaging parameters and contrast media for two cats with FHN

	Case 1	Case 2
Anaesthesia for MRI	Medetomidine^a^ 75 µg/kg IM, propofol^b^ IV isoflurane^c^, O2	Medetomidine^a^ 30 µg/kg IM, propofol^b^ IV isoflurane^c^, O2
MRI equipment and positioning	0.2 T Vet-MR, Esaote S.p.A, Genoa, Italy) with the cat placed in sternal recumbency in a human knee coil	0.2 T Vet-MR, Esaote S.p.A, Genoa, Italy) with the cat placed in sternal recumbency in a human knee coil
MRI sequences and imaging parameters and contrast media 1st examination	Pre-and post-contrast T1W trans (TR 460 ms, TE 18 ms, acquisition matrix 256 × 192, FOV 150 × 150 mm, slice thickness 4.5 mm, interslice gap 0.4 mm)	Pre-and post-contrast T1W trans (TR 750 ms, TE 26 ms, matrix 256 × 192, FOV 140 × 140 mm, slice thickness 3.5 mm, interslice gap 0.3 mm)
	T2W trans and sag (TR 2,600 ms, TE 90 ms, acquisition matrix 192 × 192, FOV 130 × 130 mm, slice thickness 4.5 mm, interslice gap 0.4 mm)	T2W trans and sag (TR 3,000 ms, TE 90 ms, acquisition matrix 256 × 192, FOV 170 × 170 mm, slice thickness 3.5 mm, interslice gap 0.3 mm)
	Gadopentet^d^ 0.1 mmol/kg IV	Gadopentet^d^ 0.1 mmol/kg IV
MRI sequences and imaging parameters and contrast media 2nd examination	Pre-contrast T1W trans (TR 750 ms, TE 26 ms, acquisition matrix 256 × 192, FOV 170 × 170 mm, slice thickness 4.0 mm, interslice gap 0.4 mm)	Pre- and post-contrast T1W trans (TR 800 ms, TE 26 ms, acquisition matrix 256 × 192, FOV 170 × 170 mm, slice thickness 4.0 mm, interslice gap 0.4 mm)
	Post-contrast T1W trans (TR 750 ms, TE 26 ms, acquisition matrix 256 × 192, FOV 170 × 170 mm, slice thickness 4.0 mm, interslice gap 0.4 mm) dors (TR 600 ms, TE 26 ms, acquisition matrix 256 × 192, FOV 180 × 180 mm, slice thickness 4.0 mm, interslice gap 0.4 mm)	
	T2W trans and sag (TR 3,000 ms, TE 80 ms, acquisition matrix 256 × 192, FOV 180 × 180 mm, slice thickness 4.0 mm, interslice gap 0.4 mm)	T2W trans (TR 3,000 ms, TE 80 ms, acquisition matrix 192 × 192, FOV 180 × 180 mm, slice thickness 4.0 mm, interslice gap 0.4 mm) and sag (TR 3,000 ms, TE 90 ms, acquisition matrix 192 × 192, FOV 170 × 170 mm, slice thickness 4.0 mm, interslice gap 0.4 mm)
	FLAIR trans and dors (TR 5,100 ms, TE 80 ms, acquisition matrix 288 × 192, FOV 220 × 220 mm, slice thickness 4.0 mm, interslice gap 0.4 mm)	FLAIR dors (TR 2,000 ms, TE 80 ms, acquisition matrix 256 × 192, FOV 200 × 200 mm, slice thickness 4.0 mm, interslice gap 0.4 mm)
	Gadopentet^d^ 0.1 mmol/kg IV	Gadopentet^d^ 0.1 mmol/kg IV

*MRI* magnetic resonance, *Trans* transverse, *Dors* dorsal, *Sag* sagittal, *FOV* field of view, *TR* repetition time, *TE* echo time, *IV* intravenously, *IM* intramuscularly.

^a^Domitor vet, Orion Pharma Animal Health, Finland.

^b^Rapinovet^®^ vet, ScheringPlough Animal Health, Denmark.

^c^IsoFlo^®^vet, Orion Pharma Animal Health, Abbot Laboratories Ltd, Great Britain; Isofluran Baxter, Baxter, Sweden.

^d^Magnevist^®^, Bayer Schering Pharma, Finland.

### Case 1

A 2 years and 2-month-old male neutered (MN) domestic shorthaired cat (DSH) was referred because of generalized tonic–clonic epileptic seizures which started 10 days prior to referral. The cat had experienced four seizures within 24 h, the last seizure had occurred within hours at the time of presentation and treatment with diazepam was given the day prior to referral. There was no previous medical history of seizures.

**Figure 1:**
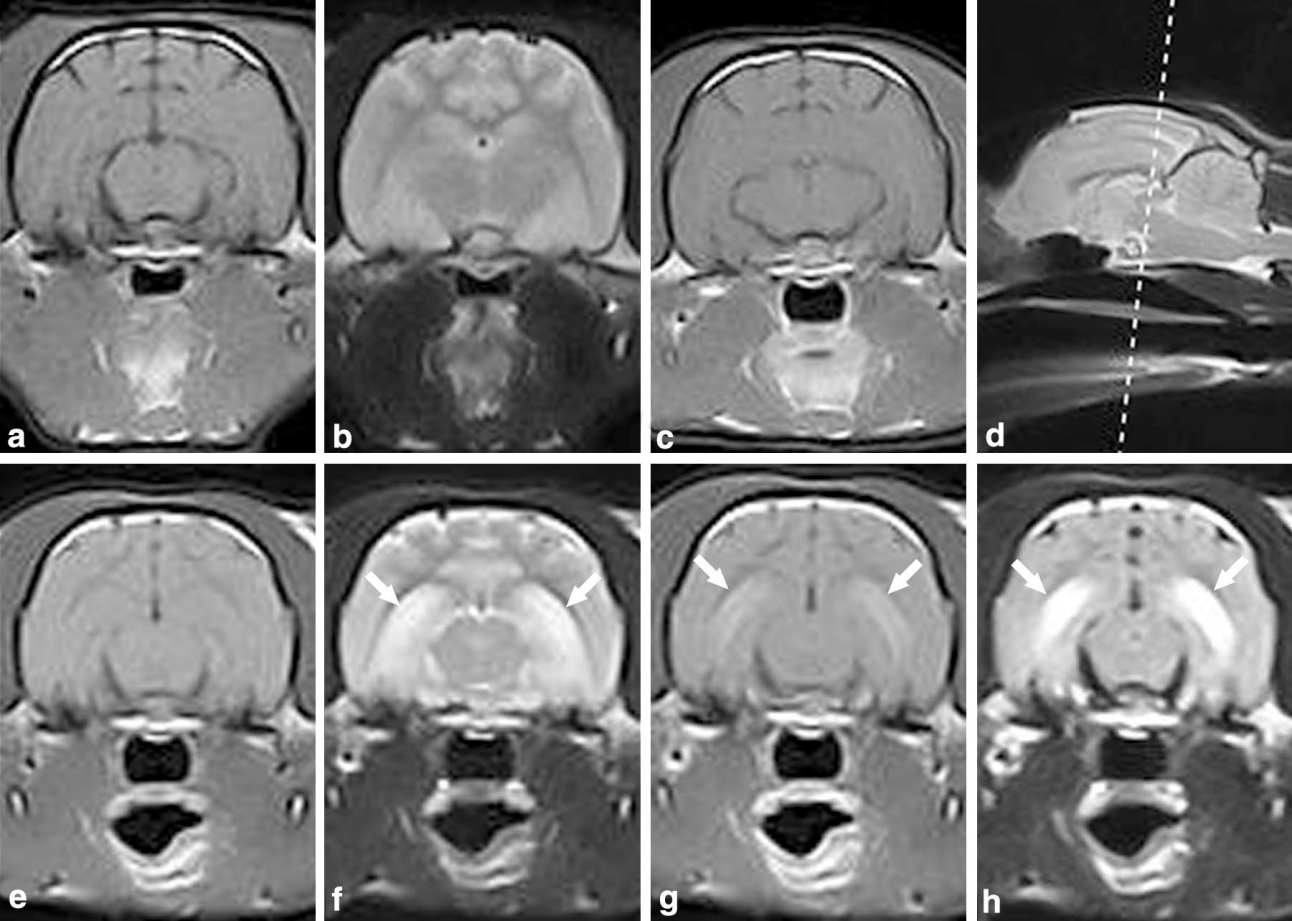
Case 1. **a**–**c** First MRI examination. A-T1W transversal, B-T2W transversal, C-T1W with contrast transversal magnetic resonance images. Normal appearance of the brain. Case 1. **e**–**h** Second MRI examination of the brain 32 months after epilepsy onset. E-T1W transversal, F-T2W transversal, G-T1W with contrast transversal, H-FLAIR transversal magnetic resonance images. *Arrows* indicate bilateral hyperintensity in the hippocampus in T2W + FLAIR and contrast enhancement in T1W + C. Magnetic resonance imaging findings consists of moderate to marked bilateral symmetric hyperintense lesions in T2W and FLAIR. Moderate to marked contrast enhancement is seen bilaterally in the hippocampus. **d** D-T2W sagittal magnetic resonance image with reference line located in rostral midbrain indicating where the transversal slices were obtained.

**Figure 2:**
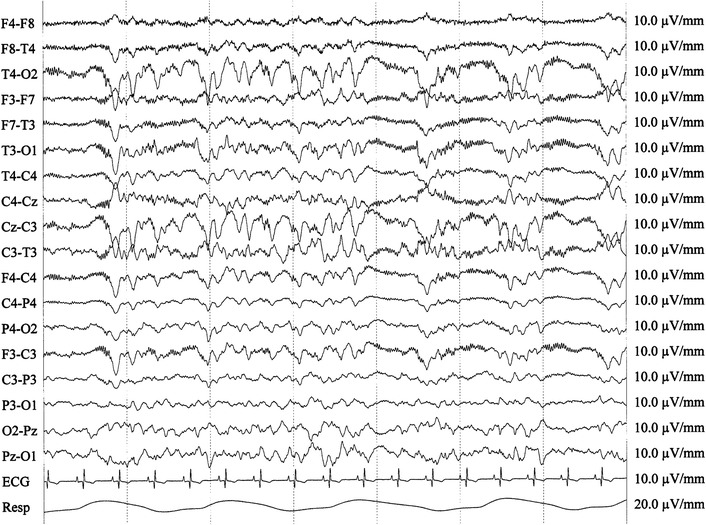
Case 1. EEG at second presentation after onset of severe cluster seizures. Continuous epileptic activity can be seen, localized at the C3 electrode, manifesting as short paroxysms and spike activity.

**Figure 3:**
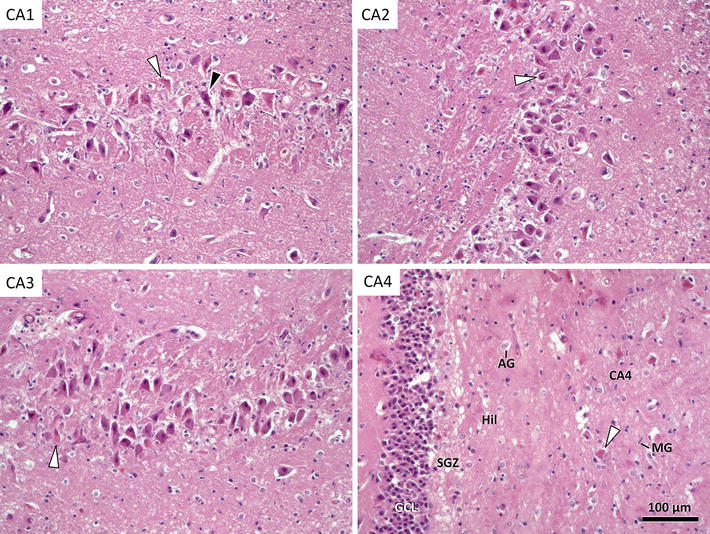
Case 1. Histopathology of the brain. Histology of different cornu ammonis segments (CA) 1 through 4. All areas show eosinophilic nerve cell necroses (*white arrowheads*). Presumable synaptic incrustation is occasionally seen (*black arrowhead*). CA4 is most severely affected and presents with severe cytodepletion next to actual nerve cell necroses. There further is an astrogliosis (AG) and microglial reaction (MG). *Hil* hilus of dentate gyrus, *SGZ* subgranular zone, *G* *CL* granular cell layer of dentate gyrus, HE, Prof.

The cat was presented in a postictal state and neurological examination revealed mild depression, abnormal postural reactions in all four limbs and decreased menace response bilaterally. Magnetic resonance imaging of the brain was performed the same day the cat was admitted and was interpreted to be normal at this stage (Figure 1a–c). Treatment with peroral (PO) phenobarbital was started. Over the following years the cat had seizures with 2–3 months interval, usually manifesting as two or three generalized tonic–clonic seizures over a few days period. The cat was normal interictally during this time period. Two years and 8 months after the onset of epilepsy, the cat had ten generalized tonic–clonic seizures within 3 days. On the 4th day, behavioural changes manifesting as aggression developed, and the owner observed bilateral mydriasis. The cat was re-evaluated at the referral clinic approximately 24 h after the last seizure. Neurological examination was difficult to perform due to aggression. Mild ataxia and tetraparesis was observed as well as exaggerated menace response bilaterally. The cat was sedated with medetomidine, 75 µg/kg intramuscularly (IM), for electroencephalography (EEG). Continuous epileptic activity localized at the C3 electrode, localized caudo-dorsally and centrally over the brain, representing the left occipital lobe, manifesting as short paroxysms and spike activity was recognized (Figure 2). Phenobarbital was given intravenously (IV) but epileptic activity persisted until anaesthesia was induced with propofol IV. Follow up MRI of the brain was performed the same day. Magnetic resonance imaging findings consisted of moderate to marked bilateral symmetric hyperintense lesions in T2W and FLAIR images as well as hypointense lesions in T1W images in the hippocampus and piriform lobe without any evidence of a mass effect. Moderate to marked contrast enhancement was recognized bilaterally in the hippocampus (Figure 1e–h). The MRI results were consistent with previous MRI descriptions of cats with FHN confirmed by necropsy [3, 4, 8, 9]. The cat was euthanized; the brain was formalin-fixed, and sent to the Neuropathology Laboratory at the Animal Health Trust, Newmarket, UK, for post mortem examination. Gross inspection of the brain revealed no abnormalities. Histopathology confirmed lesions restricted to the hippocampus and piriform lobe. Lesions were most pronounced in the dentate gyrus and the pyramidal cell layers. The majority of the pyramidal cells of the hippocampus had undergone eosinophilic neuronal necrosis and there was a notable drop out of neurons with sparse reactive gliosis (Figure 3). Moderate swelling of capillary endothelial cells and mild lymphoplasmacytic and histiocytic cuffing around nearby blood vessels were evident. A few eosinophilic neuronal necroses were observed in areas of the piriform lobe. The results of histopathology indicate sub-acute lesions and were consistent with hippocampus and piriform lobe necrosis [1–3].

### Case 2

**Figure 4:**
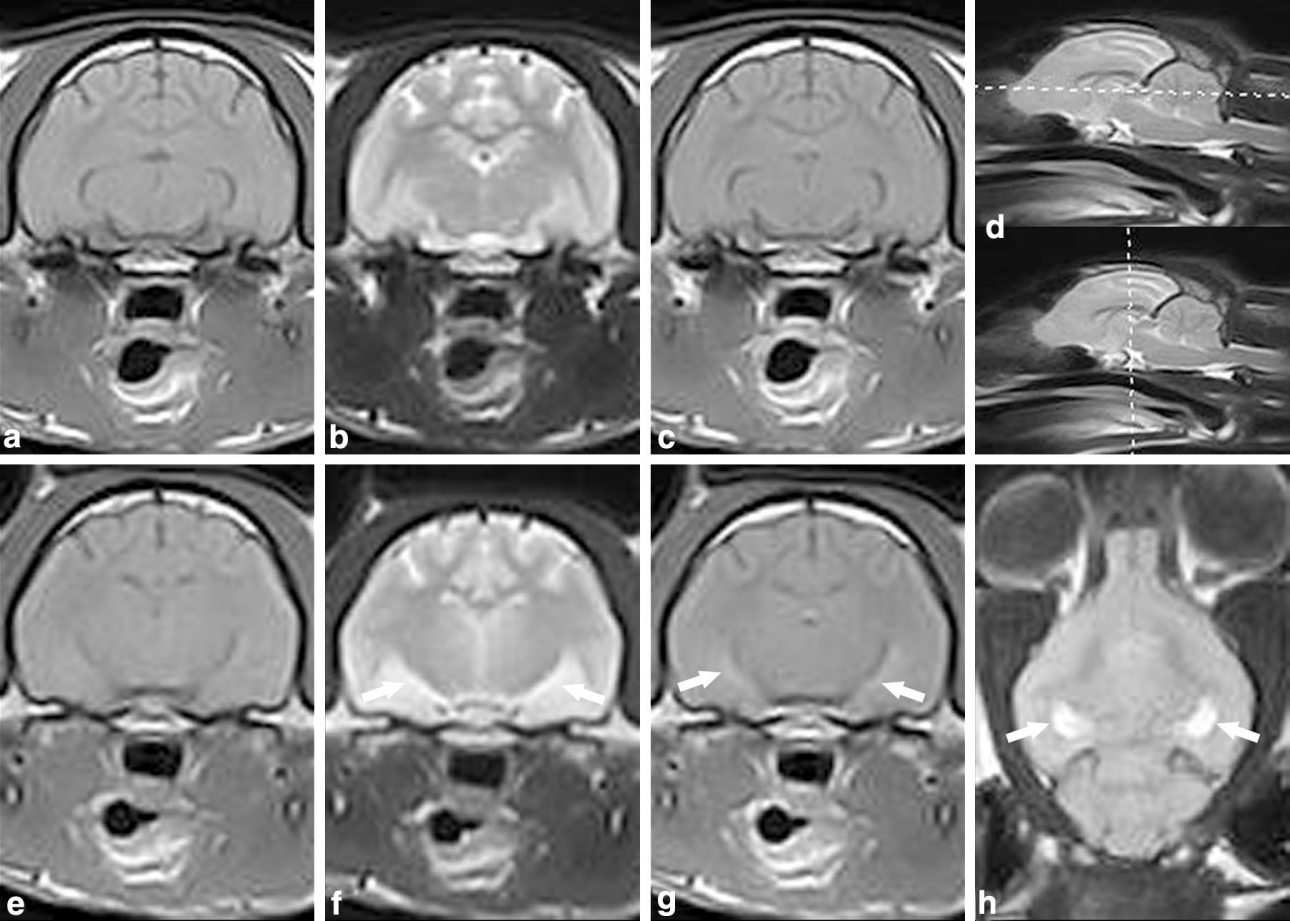
Case 2. **a**–**c** First MRI examination. A-T1W transversal, B-T2W transversal, C-T1W with contrast transversal magnetic resonance images. Normal appearance of the brain. Case 2. **e**–**h** Second MRI examination of the brain 4 days later. E-T1W transversal, F-T2W transversal, G-T1W with contrast transversal, H-FLAIR dorsal magnetic resonance images. *Arrows* indicate bilateral hyperintensity in the hippocampus in T2W + FLAIR and contrast enhancement in T1W + C. Magnetic resonance imaging findings consists of moderate bilateral symmetric T2-hyperintensity of the hippocampus. Marked bilateral hyperintensity of the hippocampus is seen in FLAIR and mild contrast enhancement bilaterally in the hippocampus in T1W post-contrast. **d** D-two T2W sagittal magnetic resonance images with one reference line located in rostral midbrain indicating where the transversal slices were obtained and one reference line showing the level where the dorsal FLAIR slice obtained.

A MN DSH cat had the first seizure episode at the age of 1 year and 2 months. The cat was examined by the referring veterinarian after the onset of epilepsy; no clinical abnormalities were found, no diagnostic imaging of the brain was performed and no antiepileptic treatment was given at this time. The cat had sporadic seizures of unspecified frequency for the next 7 months, and at this point the cat had a cluster of seizures lasting several days. The seizures were predominantly complex focal and characterized by impaired consciousness, mydriasis, salivation, aggression, piloerection of the back, twitching of the head and ears and occasionally secondary tonic–clonic generalization. The cat had seizures every 20 min the day after cluster onset, and phenobarbital therapy PO was initiated by the referring veterinarian without improvement. Three days after cluster onset, the cat was referred for evaluation and further work up. The neurological examination was performed in the postictal period and was abnormal with severe depression; postural reactions were generally decreased and menace response was absent bilaterally. During examination the cat had a short focal seizure with twitching of the face and mild salivation. Medetomidine 30 µg/kg was given immediately after the seizure for EEG examination, no epileptic activity was detected. Magnetic resonance imaging of the brain was performed after EEG and was interpreted to be normal (Figure 4a–c). The cat was hospitalized. He continued to have focal seizures repeatedly the following days, with a frequency of up to approximately one every 15 min, in spite of antiepileptic therapy with phenobarbital, diazepam and medetomidine (Table 1). The seizures were controlled after 24 h and the cat was discharged. Due to recurrent seizures, the cat was re-evaluated 4 days after the first MRI examination. The cat had the last seizure within 12 h of examination. Additional neurological signs like circling to the left and behavioural changes manifesting as severe aggression were present. A full neurological examination was not possible to accomplish. Follow up MRI of the brain was performed immediately after the neurological examination.

Magnetic resonance imaging findings consisted of moderate bilateral symmetric T2 hyperintensity and T1-hypointensity of the hippocampus and piriform lobe, no mass effect was evident. Marked bilateral hyperintensity of the hippocampus was recognized in FLAIR and mild contrast enhancement bilaterally in the hippocampus in T1W post-contrast, in accordance with a presumed ante-mortem diagnosis of FHN (Figure 4e–h) [3, 4, 8, 9]. The cat was euthanized. Necropsy was declined by the owner and not performed in this case.

## Conclusions

The two cats described here constitute the first reported cases of FHN from Finland. This verifies that FHN also occurs in Finland and should be considered as a differential diagnosis in cats with epilepsy. The clinical presentation with acute onset of severe cluster seizure (CS) activity, abnormal menace responses, abnormal behaviour and abnormal mentation, together with the results of follow-up MRI of the brain as well as the neuropathological findings reported here are identical to what has been reported earlier in cats with necrosis of the hippocampus and piriform lobe [1–3, 8, 9]. However, both of the cats reported here had a well-documented history of pre-existing sporadic seizures, of 32 months and 7 months duration respectively, before onset of severe CS activity. In case 1 the MRI done at the initial examination at seizure onset was normal, but when the cat returned with severe cluster seizures after 32 months the MRI was consistent with hippocampal and piriform lobe pathology, and the diagnosis of FHN was confirmed by histopathology. In case 2 no MRI was performed at the referring clinic at the first onset of sporadic seizures, but at the first consultation in AISTI 7 months later the initial MRI was interpreted as normal. The second MRI 4 days later after onset of severe seizure activity indicated hippocampal pathology, possibly FHN. This suggests that in both cats hippocampal pathology might have been secondary to severe seizures, although it cannot be excluded that FHN was already present at the first MRI, and may have progressed over time during the course of seizure disease, and lesions were too subtle for detecting initially. As the MRI findings are not exclusive for FHN: oedema, perfusion change, vasodilatation and blood–brain barrier alteration could not be ruled out as possible lesions leading to the described MRI changes, especially in absence of histopathology. In case 2 no post-mortem examination was allowed and the presumed FHN could not be confirmed by histology.

FHN has for a long time been accepted as a cause of seizures in cats, with a presumable environmental or toxic aetiology [1–3]. Later reports have described FHN together with neoplasia, a so called dual-pathology [10] or as a consequence of auto-immune inflammation (limbic encephalitis) [9]. Recently, FHN as a consequence of seizures has been hypothesized [5, 8]. To the authors’ knowledge, these are the first reports of cats with FHN having a normal MRI at onset of sporadic seizures and abnormal MRI at follow-up after experiencing severe cluster seizures indicating these seizures, as a possible cause for the development of subsequent FHN.

A proposed pathological mechanism preceding hippocampal necrosis is endogenous or exogenous excitotoxicity, a pathological process where neurons are damaged and/or killed by glutamate, the main excitatory neurotransmitter in the brain [13–15], or related substances. The consequence of excitotoxicity-mediated neuronal injury is selective hippocampal neuronal loss of primarily CA1 and CA3 pyramidal cells. None of the cats described in this report had any evidence of exposure to toxins. They lived primarily indoors, and other cats in the same household, exposed to the same environmental factors, remained healthy throughout the observation period. This suggests that environmental toxins are a less likely cause of FHN in the animals described here. In addition excessive glutamate concentrations and secondary endogenous excitotoxicity-mediated neuronal injury can be seen as a consequence to incidences such as head trauma, ischemia/hypoxia and hypoglycaemia [13–15]. No evidence of any traumatic, ischemic/hypoxic event or hypoglycaemia was present in the two cats making those factors unlikely to be the cause of FHN.

Dual pathology is a situation where FHN is accompanied by an additional extra-hippocampal brain lesion acting as an epileptic focus causing hippocampal sclerosis, or co-existing with the hippocampal lesion. There is one case report in the literature of a cat with FHN and an oligodendroglioma in the piriform lobe [10]. Dual pathology cannot be entirely ruled out in the cats described here but it is unlikely that a second lesion would go undetected by MRI and/or necropsy in both cases.

Suspected autoimmune limbic encephalitis (LE) associated with voltage-gated potassium channel complex antibodies resulting in hippocampal pathology in 14 cats with FEPSO-HN was recently described [9, 16]. Necropsy was available in one cat and lesions consisted of hippocampal necrosis [9]. The triggering factor for the immune-mediated process is under investigation. A positive response to immunosuppressive doses of prednisolone and antiepileptic therapy was noted in the majority of cases with full remission of signs. In case 1, LE cannot be completely excluded due to mild lymphoplasmacytic and histiocytic cuffing around blood vessels, a feature that has been described in previous reports of FHN [1–3]. No additional testing for auto-antibodies was available at the time of the case. Whether LE could be present or not in case 2 is not possible to discuss in the absence of post-mortem examination.

Necrosis of the hippocampus is described in humans [17–19] and in dogs [20–25] as a sequela to status epilepticus and/or cluster seizures, with both typical MRI changes and histopathology resembling the changes reported for cats with confirmed FHN [3, 4, 8, 9]. A well-known dilemma in humans is whether brain lesions in the postictal period are the cause or the consequence of the seizures. In humans it is also known that seizures or status epilepticus also can induce reversible, completely or incompletely, abnormalities in the brain [26–28]. These matters has not been analysed in cats but it must be taken into consideration that the same situation may be true. Cases 1 and 2, although having normal first MRI at onset of cluster seizures, showed those typical hippocampal changes on MRI later in the course of the disease after worsening of CS, indicating that lesion may be seizure-induced but it cannot be excluded that both cats may have developed FHN for unrelated reasons. Also, histological findings in case 1 were consistent with what has been described earlier in FHN and in seizure-induced lesions reported in humans and dogs [17–25, 29].

There is only limited data available on EEG in cats with naturally occurring seizures. Brauer et al. [30] did find generalized spikes as an EEG abnormality at maximum voltage in one of three cats with a presumptive diagnosis of hippocampal necrosis. Pakozdy [31] described synchronous discharges in a cat with seizures due to limbic encephalitis. The changes started in the occipital region and spread to the ipsilateral frontal and contralateral occipital region, presumably representing a subclinical partial epileptic seizure. Interestingly a convincing association between ictal semiology and limbic localization could not be confirmed by EEG in case number 1. Epileptic activity in this cat was recognized in the C3 region; placed caudo-dorsally and centrally over brain, representing the left occipital lobe, as the placement of electrodes was done resembling the 10–20 international system for humans, and not over the temporal-parietal region. This indicates that seizures might have been originating from an extra-hippocampal region, and could strengthen the hypothesis that the hippocampal changes are consequence and not cause of the seizures, although the representation of the superficial EEG may differ from the epileptic activity of deeper situated structures depending on the propagation of the seizure.

Feline hippocampal necrosis appears to have a heterogeneous aetiology and may be the consequence as well as the cause of seizures in some individuals. Our findings that cats with FHN can have a longer history of seizures preceding acute cluster seizures or status epilepticus and subsequently develop FHN, could lead to the conclusion that intense seizure activity can lead to FHN in some cats. A possible explanation why FHN as a consequence of seizures has not been reported earlier may be that cats in general are less susceptible to seizure-induced neuronal injury than other species [32]. However, ischemic injury compatible with the changes found in cats with FHN are found in experimental global cerebral ischemia [33]. Another potential reason why previously reported cases of FHN have no history of seizures preceding development of hippocampal necrosis may be that the first (often focal) seizures have not been witnessed by the owner, as many cats are living partly outdoors and/or without constant supervision. The accuracy of history can be low in such cases and cats may therefore falsely been classified as healthy prior to/until the diagnosis of FHN. A third reason might be the lack of initial and/or follow up MRI in the majority of feline patients.

Our findings that cats with FHN can have a longer history of seizures preceding acute CSs or SE and subsequent development of FHN suggest that intense seizure activity can lead to FHN in some cats. Cats with changes typical for FHN at necropsy and/or MRI may have a longer history with preceding epileptic seizures before onset of severe cluster seizures or status epilepticus. The presentation, results of MRI and histopathology suggest that cats may develop FHN secondary to severe seizure activity. Feline hippocampal necrosis occurs in Finland and should be considered a differential diagnosis in epileptic cats.

## Consent

Written informed consent was obtained from the owners of the cats for publication of this case report and any accompanying images. A copy of the written consents is available for review by the Editor-in-Chief of this journal on request.

## References

[1] Fatzer R, Gandini G, Jaggy A, Doherr M, Vandevelde M (2000). Necrosis of hippocampus and piriform lobe in 38 domestic cats with seizures: a retrospective study on clinical and pathologic findings. J Vet Intern Med.

[2] Brini E, Gandini G, Crescio I, Fatzer R, Casalone C (2004). Necrosis of hippocampus and piriform lobe: clinical and neuropathological findings in two Italian cats. J Feline Med Surg.

[3] Schmied O, Scharf G, Hilbe M, Michal U, Tomsa K, Steffen F (2008). Magnetic resonance imaging of feline hippocampal necrosis. Vet Radiol Ultrasound.

[4] Pakozdy A, Leschnik M, Sarchahi AA, Tichy AG, Thalhammer JG (2010). Clinical comparison of primary versus secondary epilepsy in 125 cats. J Feline Med Surg.

[5] Marioni-Henry K, Monteiro R, Behr S (2012). Complex partial orofacial seizures in English cats. Vet Rec.

[6] Gelberg HB (2013). Sudden behavior change in a cat. Vet Pathol.

[7] Adagra C, Piripi SA (2014). Hippocampal necrosis in a cat from Australia. Case Rep Vet Med.

[8] Pakozdy A, Gruber A, Kneissl S, Leschnik M, Halasz P, Thalhammer JG (2011). Complex partial cluster seizures in cats with orofacial involvement?. J Feline Med Surg.

[9] Pakozdy A, Halasz P, Klang A, Bauer J, Leschnik M, Tichy A (2013). Suspected limbic encephalitis and seizure in cats associated with voltage-gated potassium channel (VGKC) complex antibody. J Vet Intern Med.

[10] Vanhaesebrouck AE, Posch B, Baker S, Plessas IN, Palmer AC, Constantino-Casas F (2012). Temporal lobe epilepsy in a cat with a piriform lobe oligodendroglioma and hippocampal necrosis. J Feline Med Surg.

[11] Altay UM, Skerrit GC, Hilbe M, Ehrensperger F, Steffen F (2011). Feline cerebrovascular disease: clinical and histopathologic findings in 16 cats. J Am Anim Hosp Assoc.

[12] Jurk IR, Thibodeau MS, Whitney K, Gilger BC, Davidson MG (2001). Acute vision loss after general anesthesia in a cat. Vet Ophthalmol.

[13] Meldrum BS (2000). Glutamate as a neurotransmitter in the brain: review of physiology and pathology. J Nutr.

[14] Mark LP, Prost RW, Ulmer JL, Smith MM, Daniels DL, Strottmann JM (2001). Pictorial review of glutamate excitotoxicity: fundamental concepts for neuroimaging. AJNR Am J Neuroradiol.

[15] Silvagni PA, Lowenstine LJ, Spraker T, Lipscomb TP, Gulland FMD (2005). Pathology of domoic acid toxicity in california sea lions (*Zalophus Californianus*). Vet Pathol.

[16] Pakozdy A, Halasz P, Klang A (2014). Epilepsy in cats: theory and practice. J Vet Intern Med.

[17] Wasterlain CG, Fujikawa DG, Penix L, Sankar R (1993). Pathophysiological mechanisms of brain damage from status epilepticus. Epilepsia.

[18] DeGiorgio CM, Tomiyasu U, Gott PS, Treiman DM (1992). Hippocampal pyramidal cell loss in human status epilepticus. Epilepsia.

[19] Fujikawa DG, Itabashi HH, Wu A, Shinmei SS (2000). Status epilepticus-induced neuronal loss in humans without systemic complications or epilepsy. Epilepsia.

[20] Andersson B, Olsson SE (1959). Epilepsy in a dog with extensive bilateral damage to the hippocampus. Acta Vet Scand.

[21] Montgomery DL, Lee AC (1983). Brain damage in the epileptic beagle dog. Vet Pathol.

[22] Yamasaki H, Furuoka H, Takechi M, Itakura C (1991). Neuronal loss and gliosis in limbic system in an epileptic dog. Vet Pathol.

[23] Mellema LM, Koblik PD, Kortz GD, LeCouteur RA, Chechowitz MA, Dickinson PJ (1999). Reversible magnetic resonance imaging abnormalities in dogs following seizures. Vet Radiol Ultrasound.

[24] Hasegawa D, Fujita M, Nakamura S, Takahashi K, Orima H (2002). Electrocorticographic and histological findings in a shetland sheepdog with intractable epilepsy. J Vet Med Sci.

[25] Hasegawa D, Nakamura S, Fujita M, Takahashi K, Orima H (2005). A dog showing Klüver–Bucy syndome-like behavior and bilateral limbic necrosis after status epilepticus. Vet Neurol Neurosurg J.

[26] Cianfoni A, Caulo M, Cerase A, Della Marca G, Falcone C, Di Lella GM (2013). Seizure-induced brain lesions: a wide spectrum of variably reversible MRI abnormalities. Eur J Radiol.

[27] McNamara JO (1994). Cellular and molecular basis of epilepsy. J Neurosci.

[28] Jefferys JGR (1999). Hippocampal sclerosis and temporal lobe epilepsy: cause or consequence?. Brain.

[29] Briellman RS, Wellard RM, Jackson GD (2005). Seizure-associated abnormalities in epilepsy: evidence from MR imaging. Epilepsia.

[30] Brauer C, Kästner SB, Kulka AM, Tipold A (2012). Activation procedures in the electroencephalograms of healthy and epileptic cats under propofol anaesthesia. Vet Rec.

[31] Pakozdy A, Glantschnigg U, Leschnik M, Hechinger H, Moloney T, Lang B (2014). EEG-confirmed epileptic activity in a cat with VGKCcomplex/LGI1 antibody-associated limbic encephalitis. Epileptic Disord.

[32] Poncelet L (2011). Epileptic brain damage in dogs and cats: myth or reality?. Vet Rec.

[33] Schmidt-Kastner R, Ophoff BG, Hossmann KA (1990). Pattern of neuronal vulnerability in the cat hippocampus after one hour of global cerebral ischemia. Acta Neuropathol.

